# A Study of Young Chinese Intentions to Purchase “Online Paid Knowledge”: An Extended Technological Acceptance Model

**DOI:** 10.3389/fpsyg.2021.695600

**Published:** 2021-06-21

**Authors:** Aiting Xu, Wei Li, Zhiyu Chen, Shouzhen Zeng, Llopis-Albert Carlos, Yuhan Zhu

**Affiliations:** ^1^School of Statistics and Mathematics, Zhejiang Gongshang University, Hangzhou, China; ^2^School of Business, Ningbo University, Ningbo, China; ^3^Instituto Universitario de Ingeniería Mecánica y Biomecánica (I2MB), Universitat Politècnica de València – Camino de Vera s/n, Valencia, Spain

**Keywords:** purchase intention, online paid knowledge, technology acceptance model, perceived risk theory, cross-cultural psychology, structural equation model

## Abstract

Under the catalysis of knowledge anxiety and cognitive surplus, knowledge sharing platforms has experienced rapid growth, which has accelerated the integration of knowledge resources online. As with all new successful and sustainable business products, the consumers will play an important role in the future development of online paid knowledge. Therefore, we constructed an extended technological acceptance model by examining factors that influence young Chinese people's willingness to pay for online paid knowledge. The study surveyed 405 young Chinese participants, in which the extended technological acceptance model was tested by using structural equation modeling. Findings indicate that perceived ease of use is positively associated with perceived usefulness and associated attitudes, whereas perceived usefulness and attitude is positively associated with purchase intention. Perceived risk and group conformity are found to indirectly affect consumers' willingness to pay. The study advances the current body of knowledge by empirically testing the impact of perceived risk and the role of cultural influence (group conformity) on purchasing intention. Finally, theoretical and practical implications are discussed.

## Introduction

The rapid development of information technology has led to diverse knowledge sharing platforms that have themselves experienced rapid growth, which has accelerated the integration of knowledge resources online. These platforms provide opportunities to help people solve problems, develop new ideas in a collaborative environment, access a large pool of people with relevant knowledge, and enable cross-cultural exchange (Damian and Moitra, 2006; Kirk and Macdonell, 2014). Sharing knowledge in an open network has become a global trend, attracting not only commercial interests but also the need for further scholarly enquiry to better understand this burgeoning phenomenon (Hau and Kang, 2016; Ogundeinde and Ejohwomu, 2016).

In China, the advancement of mobile payment technology and the increasing demand for social knowledge has led to a rapidly growing number of users and the development of new products. For example, Zhihu in China (similar to Quora in the west) promotes Zhihu and Zhihu Live; Sina Weibo (Similar to Twitter) launched a product called Weibo Q&A, which provided paid written answers for its users; and Himalaya FM (similar to Spotify) began paid subscriptions for its users, which provides an extensive library of audio courses. Currently, most of these knowledge sharing platforms have sought to provide new services in order to monetize their services by charging their users (now customers) for access to particular information. Users on these platforms not only browse other users' messages for free, but also have multiple identities. They can be knowledge contributors/providers who provide professional content in related fields for free or for a fee; they can also be knowledge consumers who themselves pay for knowledge to questions they have or information they seek. Thus, knowledge platforms not only provide unpaid knowledge, but also high-quality professional fee-based knowledge. This transformation signals the importance of the knowledge purchasing industry, which has created new challenges and opportunities for both businesses and government. Statistics indicate that the scale of the knowledge purchasing industry in China reached RMB 4.9 billion in 2017, and was projected to increase to RMB 39.2 billion in 2020. It is clear that Online Paid Knowledge (OPK) is becoming a new engine for the development of China's national economy. Nevertheless, the share that this occupies in China's GDP is still relatively low compared to Europe and the United States (iResearch, 2020). More importantly, since the outbreak of COVID-19, people have been constrained from going out and communicating face to face, leading to information asymmetry. Online knowledge exchange has several advantages including cost-effectiveness, timely update, flexibility to time and place, and accessibility in gaining information (Alqudah et al., 2020; Ibrahim et al., 2020). Research indicates (e.g., iResearch, 2020) that yound people have a strong demand for gaining information for their study and work through OPKs. The increased demand for OPK represents an important new business opportunity, however, there are several challenges that it faces.

As with all new business products, the user/customer/consumers, will play an important role in the future development of OPK. Understanding the factors that motivate consumers to adopt and purchase knowledge online will assist knowledge contributors/providers and platforms to better configure their content and to provide it as quickly as possible (Litvin et al., 2008). Statistics indicate that the number of users in the knowledge purchasing industry reached 360 million in 2019, and that 82% of them stated that they would pay for quality content. Among these consumers, more than half are under the age of 30, so they are young and generally highly educated. Approximately 75.0% of users in China possess a bachelor degree or above and are willing to pay for quality knowledge content (iResearch, 2020). However, if the sharing knowledge industry is to continue to grow and proposer, consumers have to be motivated and willing to pay for it (Cai et al., 2020). Recent surveys show that only 41.0% of users are satisfied with OPK purchases, and so the repurchase rate is much lower. This raises an important question: what steps can be taken to increase consumers levels of OPK satisfaction to ensure continued purchases in the future?

To solve these problems, this study analyzes the influencing factors that affect the young generation's willingness to pay for knowledge. First, this study draws upon a new framework of TAM to measure the users'/consumers' acceptance of OPK. In addition, given the uncertainty of the network and the influence of different cultural values, we expand the TAM by combining the perceived risk theory and group consistency to understand consumers' motivation to pay. Moreover, this study strengthen the comparative analysis of the original model and the new model framework to prove the rationality and effectiveness of the proposed method. This can help us confirm that the expanded Tam is suitable for the Chinese context. In addition, examine the indirect effects of perceived risk, group consistency, perceived ease of use, and perceived usefulness on purchase intention with structural equation model. By taking this approach we argue that this study provides a deeper explanation of the complexity of consumers' purchase decisions.

**Figure 1:**
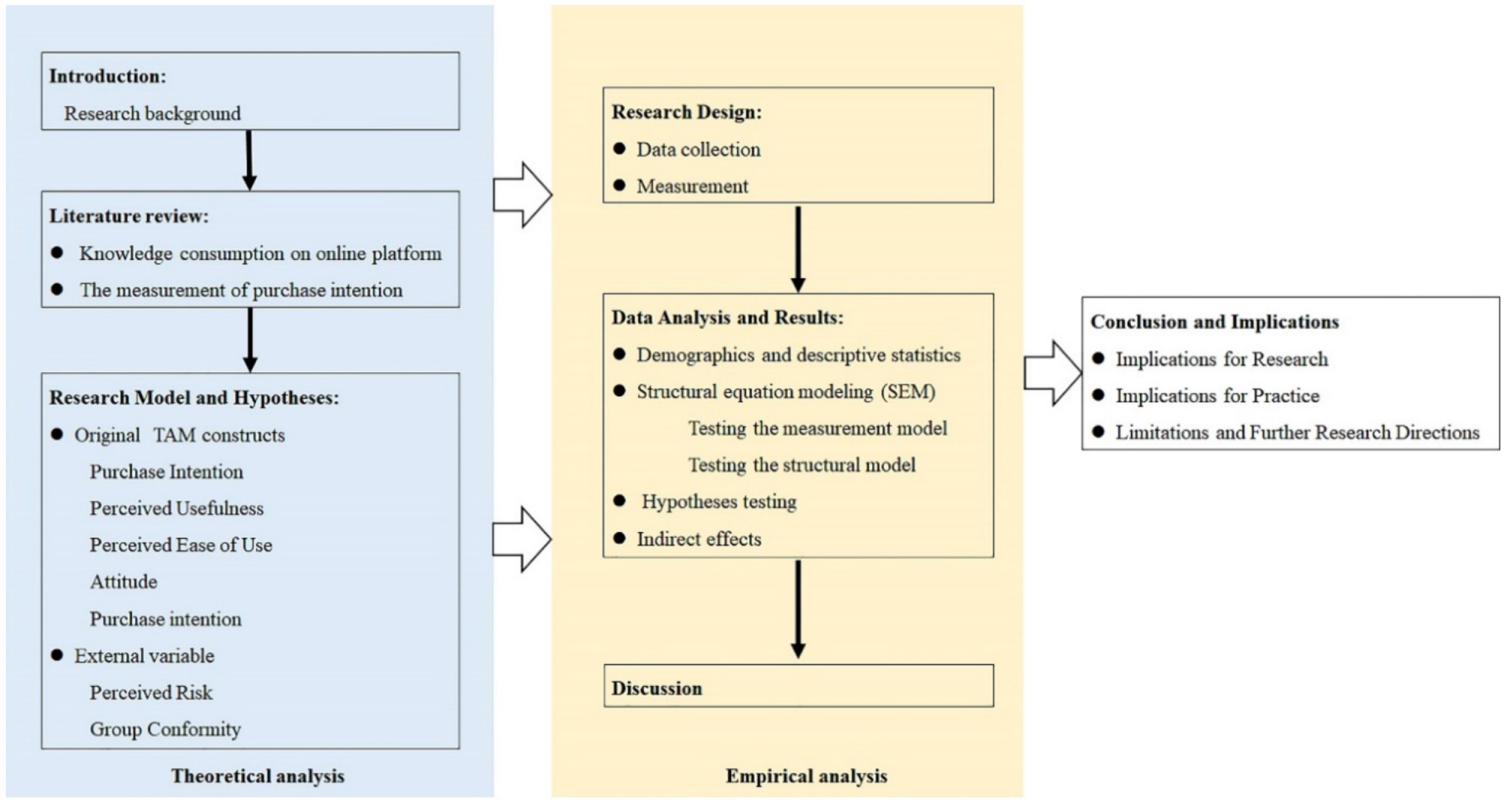
The frame diagram of the research work.

For this purpose, the work is divided into seven parts. After this introduction (1), Literature review on knowledge payment platform and the measurement of purchase intention are presented (2). In the next section, Extended TAM and theoretical hypothesis are described (3). Next, the research design (4), data analysis and results (5) are presented. Finally, the discussion (6), the conclusion, and limitations (7) are described. Moreover, the frame diagram of the research work was adopted in Figure 1.

## Literature Review

### Knowledge Consumption on Online Platform

The rapid development of internet technology has fundamentally changed the way people gain information as well as shifting consumption patterns from traditional bricks-and-mortar markets to online platforms. Knowledge sharing platforms are defined as virtual spaces where individuals can ask and answer questions of their choice through text-based computer communications so as to meet their needs for particular knowledge (Hwang et al., 2015). As for OPK, its consumption is driven by either knowledge providers or customers. For platforms requiring payment, knowledge providers offer specific knowledge in the form of text-based answers, digital audio, tutorials, and videos. OPK platforms provide a solution for users to gain necessary knowledge while providers are paid for offering their expertise.

The current literature suggest there are several benefits to OPK. Wei et al. (2015) state that OPK is often conducted by knowledgeable individuals, who are able to answer questions and provide services through their specific knowledge, skills, and experience. Cai et al. (2020) indicate that OPK covers a variety of knowledge domains related to study, work, livelihood, wealth management, and social activities, which can contribute by helping people to deal with issues from their professional and personal lives. However, high prices and content that is inappropriate or does not answer the stated question can reduce consumers' future purchase intention.

Although online knowledge sharing platforms have been widely accepted, they face several challenges. These include some of which are related to online knowledge platforms in general and those that relate to OPK platforms more specifically. Lai et al. (2012) find that knowledge systems are technological systems whereby information is integrated with those that possess a degree of expertise, and where network members exchange information with each other. While lower barriers to access knowledge systems facilitate a wider range of contributions, they can also result in low-quality information and misinformation (Shao et al., 2018), which can lead to information redundancy. In addition, the perceived quality of OPK is generally influenced by the customers' level of expertise. So that customers with lower levels of knowledge may not be able to accurately judge the quality and authenticity of the information they are receiving. Thus, intellectual differences and subjective judgement will influence the users views toward online knowledge. Furthermore, with an increasing number of people using knowledge payment platforms, privacy is another important issue that these online services need to tackle. In particular, the need to protect individuals and corporate private information from falling into the hands of a third party, has become a controversial issue in the online world (Li et al., 2018). These difficult to manage and uncertain features of the online knowledge product make customer purchase intention even more complex to understand. The existing research on knowledge platforms has also focused on their profitability and the number of knowledge platforms that now inhabit the marketplace (Cai et al., 2018), knowledge management strategies (Karimi et al., 2015), and further development of the knowledge intensive industry (Lee and Jung, 2020). At this stage few studies have sought to examine users' willingness to adopt and pay for OPK, which is a key issue if these businesses are to survive and prosper. Therefore, our study employs the TAM, which will be augmented for the first time with two new contructs—*perceived risk* and *group conformity*—to analyze the determinants of young Chinese consumers' purchase intentions (Ma and Chan, 2014).

### The Measurement of Purchase Intention

Scholars have identified a variety of factors that influence people's online purchase behaviors. Hölscher and Strube (2000) find that too much online information made the consumer purchase decision-making process more complicated and onerous. Meanwhile, research suggests that potential factors like usefulness, ease of use, trust, perceived risk, attitudes, social influence, and subjective norms are critical factors in the consumer decision process when it comes to online payment systems (Hausman and Siekpe, 2009). Among these factors, perceived risk plays an important role in the process of purchase decision-making. However, few studies have focused on online knowledge sharing platforms, in which customers have higher expectations on the value of the knowledge they receive. At the same time, users take a potential risk in gaining information that is redundant, inappropriate or not useful while being overcharged for it (Zhang et al., 2019). Users also show concerns over the privacy of the information they obtain. Thus, we introduce *privacy* as a major factor in our research.

Despite the increasing concerns regarding online purchase risks, the literature is limited in this respect, and the research that does exist concentrates on the different nations and their diverse cultural environments, which could lead to differences in consumption values (Gârdan et al., 2016). Pena-Garcia et al. (2020) indicate that there is an intimate connection between culture and consumption behavior. Aaker et al. (2001) analyze consumption symbols from a psychological perspective in a cross-cultural context, showing that national cultural influence is significant. We also consider cultural differences/influences to be an important factor in the Chinese context.

The existing research has also considered and applied various theories designed to predict consumer purchase intention in the online context, such as the Theory of Reasoned Action (TRA) (Fishbein and Ajzen, 1975), the Theory of Planned Behavior (TPB) (Ajzen, 1991), and the Technology Acceptance Model (TAM) (Davis, 1989). Among these theories, the TAM has been one of the most widely and successfully employed in the various contexts of information services, such as mobile app technology, online shopping websites, and e-procurement systems. The original TAM includes determinations about behavior intention including perceived ease of use and perceived usefulness to evaluate the adoption of new information systems, which have extended the TAM in its application to the technology-driven context. Apart from the original construct, prior studies have attempted to add external factors to the TAM to fit different contexts (Lee, 2009; Zeng et al., 2021). Thus, this research not only applies but also extends the TAM by applying it to both intrinsic and extrinsic constructs to study OPK.

## Research Model and Hypotheses

### The Amendment and Extension of Technology Acceptance Model

The TAM was first proposed by Davis (1989). It was considered to be one of the most influential theories to explain the acceptance and usage of information technologies. The model assumes that perceived usefulness and ease of use are primary determinants of users' decisions, which will affect their attitude toward the behaviorial intention (Davis et al., 1989). However, Legris et al. (2003) stated that the TAM only explained 40.0–60.0% of consumer behavior intentions. To enhance the explanatory power of the TAM in a specific settings, existing studies have begun to employ a multifaceted approach to its application (Lee et al., 2010).

There are three main types of extended TAM. The first type concentrates on social impact theory and perceived risk theory (Kamal et al., 2020). The second group focuses on factors acting as intermediaries (such as consumers' satisfaction, gender, age) (Baudier et al., 2020). The third group incorporates factors from other models such as perceived behavior control, and subjective norms. Moreover, recent research studys online purchase intentions to assist management to understand buyers' perspectives. Kim (2012) studied consumers' first purchase intentions in online shopping by integrating trust into the original TAM. Ramkumar et al. (2019) extended the TAM with system quality to predict buyers' continuous purchase intention for e-production services. In line with these previous studies, the current research extends the TAM by adding two external factors. One is perceived risk, adopted from perceived risk theory (Bauer, 1969) and the other is group conformity, which represents subjective norms from the Theory of Reasoned Action (TRA).

**Figure 2:**
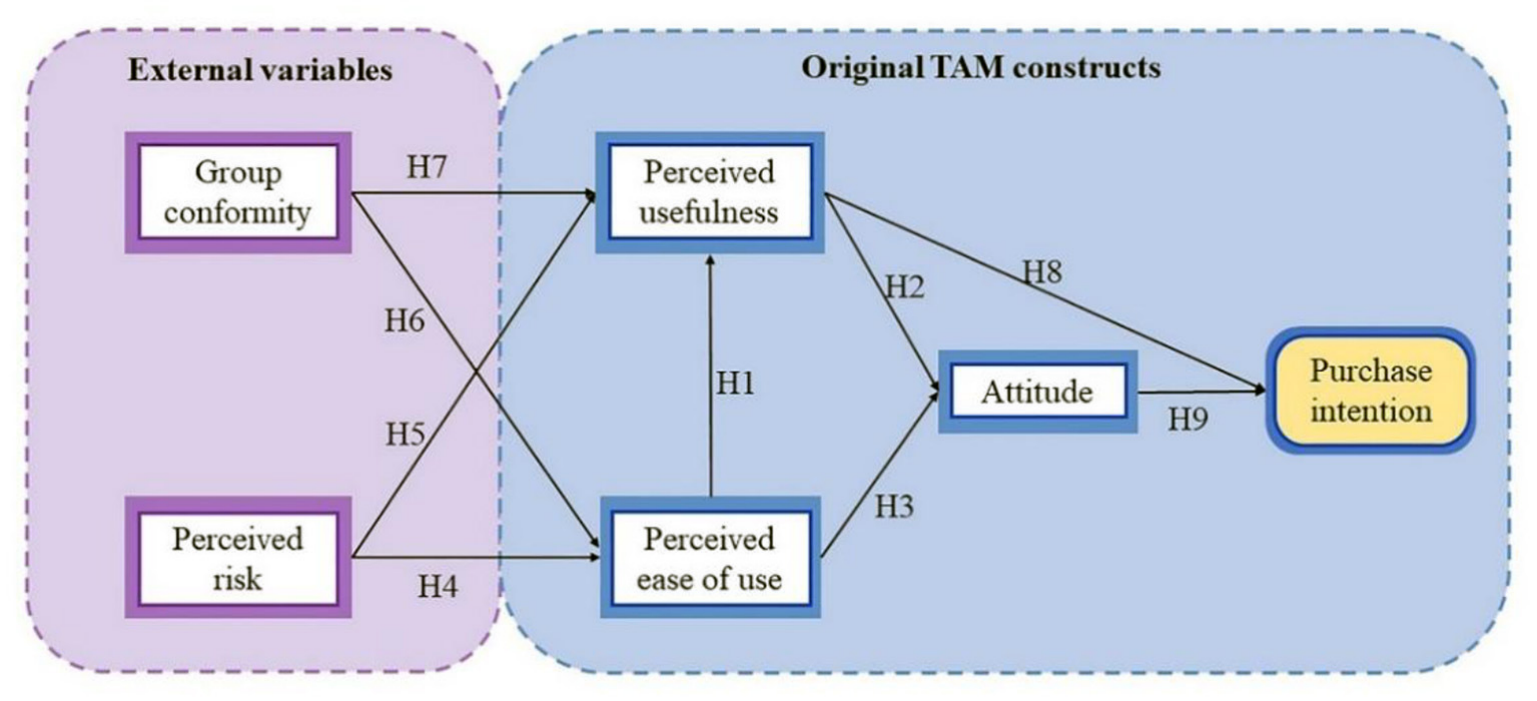
Hypothesis testing results. *p < 0.05, **p < 0.01, ***p < 0.001.

Based on the amended and extended TAM, we propose a new conceptual framework depicted in Figure 2. In the original TAM, perceived ease of use (PEOU), perceived usefulness (PU), and attitude (ATT) are posited as key predictors that influence consumers' purchase intention (PI) of OPK. Considering that cultural value differences and online purchase environment may potentially affect willingness to pay, we have added perceived risk (PR) and group conformity (GC) as the external variables in the proposed model. Further discussion on the main factors and hypotheses will be presented in the next section.

### Research Hypotheses Based on External Variables

#### Perceived Usefulness, Perceived Ease of Use and Attitude

The basic assumptions underpinning the TAM are perceived ease of use and perceived usefulness (Davis, 1989). Both of them are noted as important determinants that directly influence users' attitudes. Perceived ease of use is defined as the degree to which an individual believes that the usage of a particular technology does not require extra effort (Davis, 1989). Perceived usefulness reflects the extent to which a person believes that the use of a specific system can enhance their work performance. Prior studies have consistently reported that perceived ease of use and perceived usefulness affect users attitudes toward the adoption of information and technology systems (Scherer et al., 2019).

Prior studies of online purchasing indicate that the TAM has been widely used to explore the relationship between perceived ease of use, perceived usefulness and users' attitudes to online purchase of services or products. For instance, Agag and El-Masry (2016) argued that the TAM linked to the variable of trust offers an appropriate model for understanding consumers' intention to purchase travel online, as well as revealing that perceived ease of use and perceived usefulness had a significant positive impact on consumers' attitudes to participate in an online travel community. Meanwhile, perceived ease of use was positively associated with perceived usefulness. Law et al. (2016) provided an extended online purchase intention model by adding habitual online usage, which enhanced the TAM in the online context by improving the readability of online purchase intentions. Similarly, in this study, the online knowledge payment platform is one of the most important commercial channels in the knowledge service industry and information systems. Perceived ease of use represents the consumers' beliefs that OPK is an easy or more effortless way to gain knowledge. The more easily consumers gain information, the more positive their attitude toward OPK purchasing will be. For example, Hsu and Lin (2008) developed an integrated TAM with knowledge sharing and social influence, showing that perceived ease of use appeared to be an important motivation which enables users to participate in a knowledge community. Thus, we propose:

**H1: Perceived ease of use is positively associated with perceived usefulness**

**H2: Perceived ease of use is positively associated with attitudes**

In this study, perceived usefulness is defined as the extent to which consumers believe purchasing OPK through online platforms will bring significant value to them. Earlier research reported that consumers' attitudes toward purchases might be influenced by their judgement of the usefulness of the services they enjoyed. For example, Yang et al. (2015) investigated young Chinese consumers who were the largest demographic that paid for goods and services revealing that consumers' perceived usefulness significantly increased purchase intention in the online payment environment. Renny et al. (2013) also suggest that perceived usefulness had a significant influence on consumers' attitudes toward online shopping. Therefore, we propose:

**H3: perceived usefulness is positively associated with attitudes**

#### Perceived Risk

Perceived risk is regarded as an important factor in the online purchasing environment, which is first introduced from perceived risk theory. It describes “the possible negative consequences of purchasing a new product or service that influence the customer behavior” (Bauer, 1969). In this study, perceived risk is generally viewed as a serious obstacle that plays an important role in consumers' adoption decisions. To adapt to the virtual network environment, some researchers redefined the concept of perceived risk. Chiu et al. (2014) argued that the issues of online communication made information-based risk an uncertain factor in the e-commerce environment, including security, recommendation and personal privacy. Wu et al. (2020) proposed that perceived risk has four attributes that include financial, product, recommendation, and security in online stores. Nepomuceno et al. (2014) redefined it as the subjective expectation of loss. One of the most beneficial functions of OPK was to provide valuable and meaningful information. Therefore, considering the possibility that a consumer may receive redundant, inappropriate or less than useful knowledge, was opertionalised in this study as perceived risk, which was defined as “the possibility of losing control over one's personal information and obtaining worthless information.”

Prior empirical research has extended the TAM by integrating perceived risk by looking to explore the relationship between the central factors and external variables. For example, Hansen et al. (2018) indicated that perceived risk played a significant role as an antecedent in consumers' decision-making processes in the context of social networking sites, showing that perceived risk was associated with the rise of perceived ease of use. Featherman and Pavlou (2003) observed that perceived risk reduced perceived ease of use and perceived usefulness of e-services. Featherman and Fuller (2003) found that high levels of perceived risk redcued consumers' perspectives of ease and usefulness. Thus, we propose:

**H4: Perceived risk is negatively associated with perceived ease of use**

**H5: Perceived risk is negatively associated with perceived usefulness**

#### Group Conformity

The concept of group conformity is drawn from the cross-cultural psychology literature (Harb and Smith, 2008), representing the subjective norm from the TRA to measure cultural factors that influence purchase intention. Subject norm thus far has not been tested in generic TAM, however, it is a significant determinant of behavior intention in TRA (Ajzen, 1987). However, subjective norm is not always adequate enough to predict intention in different cultures. Given the cultural differences, the TRA and TAM have been developed and improved in the western context; however, the validity and interpretability of subjective norm in explaining consumers' intention is considered to be controversial under specific conditions. Thus, several studies have attempted to incorporate cultural values with the TAM, suggesting that different cultural systems continue to occupy an important position in the consumer decision-making process. Therefore, we amend and extend the TAM to fit the Chinese context by introducing Chinese cultural constructs by using group conformity to represent subjective norm.

In this study, group conformity refers to the degree to which an individual behavioral intention is influenced by groups, which tend to converge in their thinking and behavior (Qi and Ploeger, 2019). In China, social influence from peers, multimedia or group norms are often more influential than they are in western countries (Li and Su, 2007). Studies on the adoption of new technologies reveal that collectivism is the main reason why users adopt certain technologies from collectivist societies. The reason for this is that it is an effective and easy way for consumers to avoid losses by taking communities' suggestions into consideration. Jap (2010) suggested that Confucian culture in which the concept of face plays an important role, influences Chinese consumption patterns, which were often to satisfy the desire for an enhanced social status. Moreover, Mainolfi (2019) argued that conformity plays a powerful role in China and that it affects consumers' cognitive beliefs and judgement of their intentions to purchase online. This is particularly true for the young groups, who are the most avid consumers of online products, and who are also more easily influenced by social or peer communications. Their intention to adopt new technology is frequently influenced by the views and perceptions of their own social circle, such us friends, teachers and other related social groups. Therefore, we propose:

**H6: Group conformity is positively associated with perceived ease of use**

**H7: Group conformity is positively associated with perceived usefulness**

#### Purchase Intention

Purchase intention refers to *a consumers' degree of willingness to pay for OPK*. While perceived ease of use, perceived usefulness, and attitude are important antecedents of personal intention. Research by Davis and colleagues found that usefulness and attitude generally have the strongest influence on actual behavior (Davis, 1989). In addition, in online knowledge sharing, the quality of online knowledge can be directly measured by its value and price, which are regarded as important factors that influence consumers' attitudes to purchase (Zhang et al., 2019). In China, differences in product prices, brand names, promotions, and distribution have been found to influence a range of different attitudes. For the purpose of this study, attitude will be defined as “an individual's positive or negative judgement of the knowledge quality by its value and price.”

Existing studies focus on the purchase intention of online services or products. Chang and Chen (2008) proposed a new model based on the stimulus-organism-response framework to examine the relationship between perceived risk, trust and consumers' purchase intention. Purnawirawan et al. (2012) observed that only when information was viewed useful through the recall of a positive or negative review did it influence the users' attitudes and intention through the impression it created about the object. It is apparent that beneficial information will change consumers' attitudes and purchase intentions. Further, Gao and Huang (2019) expanded on the TAM by adding users' smart service beliefs. Their study found that perceived ease of use and attitude of users' had a direct and positive influence on consumers' intentions to accept smart services. Thus, we propose:

**H8: Perceived usefulness is positively associated with purchase intention**

**H9: Attitude is positively associated with purchase intention**

## Research Design

### Data Collection

An online survey was conducted to collect data for this study. The sample comprised college students from 20 universities in Zhejiang Province, China. The main purpose of the research was to examine the factors that influence young Chinese people's purchase intention of OPK, the rationale for selecting college students was based on two main reasons: (1) they were the main users of the Internet, and most of them had a strong tendancy to accept new technologies such as OPK (Chao et al., 2011) and (2), college students would be the main consumer group of OPK in the future.

The online questionnaire was comprised of two main sections. (1) The first half was made up of socio-demographic questions. (2) The second half was made up questions to test the proposed structural model. A pilot study was initially conducted to test the measurement instrument and determine the feasibility of the study protocol. Then the questionnaires were distributed to college students using a stratified random sampling method between February and March of 2020. Four hundred thirty questionnaires were completed, 25 of which were not fully completed. In total, 405 questionnaires were used in the subsequent analysis. All of the questionnaires were presented in Chinese.

### Measurement

Twenty-three items were included in the questionnaire to address the factors that influence the purchase intention of young Chinese people's use of OPK (Table 1). All the items used a five-point Likert scale ranging from 1= “strongly disagree” to 5 = “strongly agree.”

**Table 1:** Measurement scales.

**Construct**	**Label**	**Items**	**Adopted from literature**
Purchase Intention (PI)	PI1	I am very likely to buy the knowledge product	Davis, 1986; Gefen and Straub, 2004
	PI2	I will consider buying the knowledge product.	
	PI3	I will try to use OPK if it's necessary in life or work.	
Perceived Usefulness (PU)	PU1	OPK can increase the efficiency of my life and work.	Kim et al., 2007; Kuo and Yen, 2009
	PU2	OPK is useful in performing my task.	
	PU3	OPK improves my task performance.	
	PU4	OPK can help me accomplish tasks in my life and work more easily.	
	PU5	Using knowledge payment platform enables me to accomplish tasks more quickly.	
Perceived Ease of Use (PEOU)	PEOU1	It is convenient to find OPK what I really want.	Karahanna and Straub, 1999; Kim et al., 2007
	PEOU2	It is easy to use knowledge payment platform.	
	PEOU3	Learning to use knowledge payment platform is very simple.	
	PEOU4	I believe that it is easy to get knowledge payment platform to do what I want it to do.	
Attitude (ATT)	ATT1	Using OPK is a good idea	Taylor and Todd, 1995; Yadav and Pathak, 2016
	ATT2	Using OPK is pleasant.	
	ATT3	The choice of using OPK is wise.	
	ATT4	I am pleased with the fee that I have to pay for the use of OPK	
	ATT5	For the effort involved in OPK, paying for knowledge is worthy.	
Perceived Risk (PR)	PR1	I will worry that the payment environment of knowledge payment platform is not secure enough.	Stone and Gronhaug, 1993; Geetha and Kalyani, 2015
	PR2	I will worry that the knowledge payment platform may leak my personal information.	
	PR3	I will worry that the knowledge I get through paying doesn't meet my expectations.	
Group Conformity (GC)	GC1	I will use OPK, if people around me use OPK too.	Qi and Ploeger, 2019; Vahdat et al., 2021
	GC2	People around me have a positive attitude toward OPK.	
	GC3	I may consider using OPK, if people around me think OPK is good.	

## Data Analysis and Results

### Demographics and Descriptive Statistics

48.1% of the respondents were male (195) with 51.9% female (210). For educational background, 29.6% of respondents were juniors, 23.2% were seniors, 22.2% were freshman, 23.2% were sophomore, and 4.2% were postgraduate. The majority of respondents had monthly living expenses of CNY1001-2000 (70.1%) followed by the CNY2001- CNY3000 group (24.0%). In terms of frequency of payment for knowledge, 57% of respondents paid for knowledge no more than once a week, whilst only 6.2% of respondents paid for knowledge more than 7 times a week (Table 2).

**Table 2:** Demographic information.

**Demographic information**	**Frequency (*n*)**	**Percentage (%)**
**Sex**		
Male	195	48.1%
Female	210	51.9%
**Educational background**		
Freshman	90	22.2%
Sophomore	84	20.7%
Junior	120	29.6%
Senior	94	23.2%
Postgraduate	17	4.2%
**Monthly living expenses**		
<1,000 yuan	9	2.2%
1,001–2,000 yuan	284	70.1%
2,001–3,000 yuan	97	24.0%
More than 3,000 yuan	15	3.7%
**Frequency of payment for knowledge**		
More than 7 times a week	25	6.2%
5–7 times a week	37	9.1%
2–4 times a week	112	27.7%
No more than once a week	231	57.0%
**Where to get knowledge (Responders can choose more than one answer)**		
Books	298	73.6%
Asking people around	228	56.3%
Search engine	363	89.6%
Knowledge payment platform	265	65.4%
Others	5	1.2%
**Content preference (Responders can choose more than one answer)**		
Working skills	306	75.6%
Financial management	94	23.2%
Knowledge related to hobbies	103	25.4%
Professional knowledge	337	83.2%
Health-related knowledge	43	10.6%
Emotion-related knowledge	27	6.7%
Others	12	2.9%

In terms of content preference, professional knowledge and working skills were the ones that respondents were most willing to pay for, accounting for 83.2 and 75.6% respectively. The type of content they were less willing to purchase included knowledge related to hobbies (25.4%) and financial management (23.2%). Respondents were also reluctant to pay for health-related and emotion-related knowledge, which only accounted for 10.6 and 6.7% respectively.

### Structural Equation Modeling (SEM)

#### Testing the Measurement Model

When performing SEM, researchers first evaluate the measurement model (whether the measured variables accurately reflect the desired constructs or factors) (Doll et al., 1994), because poor measurement properties can lead to erroneous conclusions regarding the existence, magnitude, and direction of association between constructs (Segars, 1997).

We examined the reliability and validity of the measurement model using IBM SPSS 22.0 and AMOS 22.0. Table 3 outlines the Cronbach's Alpha coefficients for all of the constructs, which were higher than 0.7 signifying a good internal consistency (Koufteros, 1999). Table 3 outlines all figures above 0.7, exhibiting a good reliability (Anderson and Gerbing, 1982). The Average Variance Extracted (AVE) values were almost above 0.5, showing that the data had a good convergent validity (Koufteros, 1999).

**Table 3:** Construct reliability and convergent validity.

**Constructs**	**Items**	**Factor loading**	**CR**	**AVE**	**Cronbach's Alpha**
Purchase Intention (PI)	PI1 PI2 PI3	0.874 0.838 0.745	0.860	0.674	0.857
Perceived Usefulness (PU)	PU1 PU2 PU3 PU4 PU5	0.847 0.759 0.771 0.665 0.775	0.847	0.528	0.847
Perceived Ease of Use (PEOU)	PEOU1 PEOU2 PEOU3 PEOU4	0.791 0.765 0.691 0.685	0.803	0.506	0.803
Attitude (ATT)	ATT1 ATT2 ATT3 ATT4 ATT5	0.632 0.514 0.621 0.812 0.791	0.792	0.438	0.795
Perceived Risk (PR)	PR1 PR2 PR3	0.756 0.828 0.553	0.765	0.528	0.757
Group Conformity (GC)	GC1 GC2 GC3	0.687 0.872 0.726	0.802	0.578	0.798

Traditional methods for testing discriminant validity, such as the Fornell-Larcker criterion and cross-loadings, did not perform well in many research contexts (Henseler et al., 2014). Therefore, Henseler et al. (2014) proposed a new approach testing termed the heterotrait-monotrait (HTMT) ratio of correlations method, which had a higher sensitivity and specificity (Ab Hamid et al., 2017). When <0.90, it meant that the discriminant validity had been established (Henseler et al., 2014). As was shown in Table 4, the discriminant validity of our model was acceptable.

**Table 4:** HTMT results.

	**PI**	**PU**	**PEOU**	**ATT**	**PR**
PU	0.743				
PEOU	0.607	0.778			
ATT	0.733	0.836	0.705		
PR	0.326	0.366	0.226	0.251	
GC	0.220	0.339	0.235	0.323	0.325

#### Testing the Structural Model

To assess the model fit, we followed the recommendations on the convenience of using multiple adjustment indicators (Jackson et al., 2009). Table 5 illustrated that CMIN/DF was 2.415, which met the standard (between 1 and 3) and RMSEA was 0.059, less than the highest standard (0.08), indicating the gap between the theoretical model and the saturated model was in a reasonable range. The Goodness-of-Fit Index (GFI) was 0.892. Many researchers interpreted that GFI scores in the 0.800–0.899 as a reasonable range fit; scores of 0.900 or higher are considered to be of good fit (Doll et al., 1994). The Comparative Fit Index (CFI), Increasing Fit Index (IFI), and the Tucker Lewis Index (TLI) were 0.926, 0.925, and 0.915 respectively, which met the evaluation standard of 0.900. Therefore, the overall fit of the theoretical model was within an acceptable range (Iacobucci, 2010).

**Table 5:** Goodness of fit indices.

**Index**	**Definition**	**Index results**	**Evaluation standard**
CMIN/DF	χ^2^/*df* ratio	2.415	1–3
RMSEA	Root Mean Square Error Approximation	0.059	<0.080
CFI	Comparative Fit Index	0.926	>0.900
GFI	Goodness of Fit Index	0.892	>0.800
IFI	Incremental Fit Index	0.925	>0.900
TLI	Tucker-Lewis index	0.915	>0.900

### Hypotheses Testing

**Figure 3:**
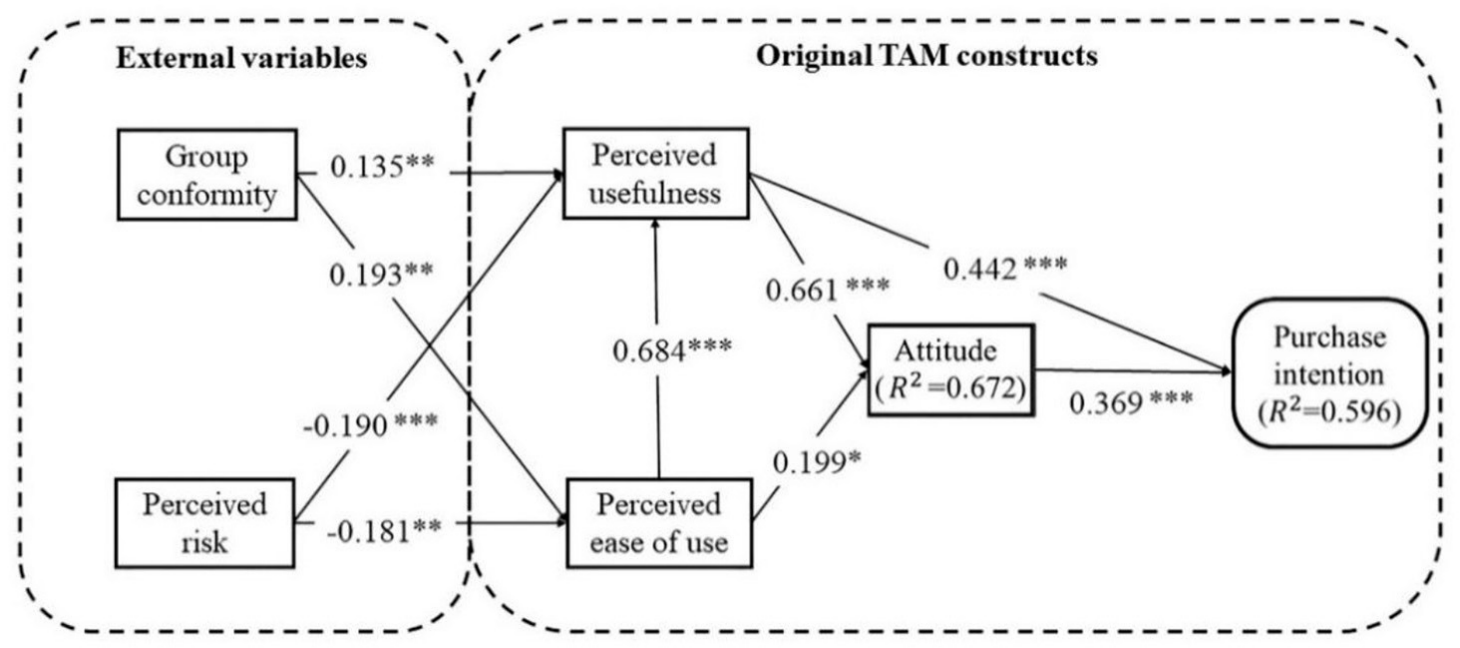
Research model.

As illustrated in Table 6, Figure 3, all the hypotheses were supported. The influence of perceived ease of use on purchase usefulness and attitudes was positive and significant, supporting H1 and H2. H3 influence of perceived usefulness on attitudes was also supported.

**Table 6:** Hypotheses of the research.

**Hypotheses**	**Relationship**	**Path coefficients**	***P*-value**	**Result**
H1	PEOU→PU	0.684	^***^	Supported
H2	PEOU→ATT	0.199	0.014	Supported
H3	PU→ATT	0.661	^***^	Supported
H4	PR→PEOU	−0.181	0.003	Supported
H5	PR→PU	−0.190	^***^	Supported
H6	GC→PEOU	0.193	0.001	Supported
H7	GC→PU	0.135	0.004	Supported
H8	PU→PI	0.442	^***^	Supported
H9	ATT→PI	0.369	^***^	Supported

*** *values are significant at p < 0.001*.

The results also confirmed the negative impact of perceived risk on perceived usefulness and perceived ease of use, supporting H4 and H5. Group conformity also had a positive influence on perceived usefulness and perceived ease of use, supporting H6 and H7. H8 and H9 were supported by the significant path coefficient of 0.442 (*p* < 0.001) and 0.369 (*p* < 0.001) respectively.

### Indirect Effects

In order to assess the significance of indirect effects on the variables related to consumers' purchase intention of OPK, bootstrapping procedures were conducted. We followed the bootstrapping method proposed by Zhao et al. (2010) based on Baron and Kenny's criteria for establishing mediation (Baron and Kenny, 1986). If the confidence interval did not include 0, the indirect effect was significant and the mediation hypotheses are supported. In this study, the number of bootstrapping resamples which were selected from the original data through a random sampling way was 2000. The standard deviation of these indirect effect sizes across the resamples was provided at the 95% confidence level.

Table 7 illustrated that all four path coefficients were significant and the confidence interval did not include 0, which means the indirect effect was significant and mediation hypotheses were supported. Although perceived risk, group conformity and perceived ease of use had no direct effects on purchase intention, they indirectly affect consumers' willingness to pay. Furthermore, perceived usefulness could affect consumers' purchase intention significantly in both direct and indirect ways.

**Table 7:** Bootstrapping indirect effects.

**Relationship**	**Independent variable**	**Dependent variable**	**Path coefficients**	**(95% CI) Bootstrapping (Lower bound-Upper bound)**
PR→PEOU, PU, ATT→PI	Perceived risk	Purchase intention	−0.228^**^	(−0.352, −0.105)
GC→PEOU, PU, ATT→PI	Group conformity	Purchase intention	0.198^***^	(0.103, 0.304)
PEOU→ATT, PU→PI	Perceived ease of use	Purchase intention	0.543^***^	(0.433, 0.631)
PU→ATT→PI	Perceived usefulness	Purchase intention	0.244^*^	(0.066, 0.439)

*N = 405*;

* *p < 0.05*,

** *p < 0.01*,

*** *p < 0.001*.

## Discussion

The results of this study indicate that extended TAM is an appropriate model for the analysis of Chinese young consumers' technological acceptance of OPK. As expected, perceived ease of use is positively associated with perceived usefulness and attitude (H1 and H2). The same is true between perceived usefulness and attitude (H3). Furthermore, perceived usefulness and attitude are positively correlated with purchase intention (H8 and H9). These results of original TAM constructs in our study are in line with what are proposed in the initial TAM (Davis, 1986), and are also consistent with the conceptual model proposed in the prior literature (Sánchez and Hueros, 2010; Zheng and Li, 2020). Thus, we can know that young people in China are more likely to buy OPK when they think it is useful and easy to use. And those who are with a more positive attitude toward OPK have stronger purchase intention of OPK.

In order to enhance the explanation ability of TAM in specific setting and fit in Chinese context, we add two new external constructs (perceived risk and group conformity). The results of this study provide supports for revealing the effects of two new constructs on the four variables of original TAM. Although perceived risk does not have a direct impact on consumers' intention to use OPK, the findings demonstrate that it can bring negative effect on perceived ease of use and perceived usefulness (H4 and H5), which finally indirectly awaken purchase intention of OPK. Thus, the reduction of perceived risk can exert a significant effect on promoting knowledge purchase intention of China's younger generation. Such results are similar to prior studies which explore the role of perceived risk (Featherman and Pavlou, 2003; Chiu et al., 2014; Wang et al., 2019).

Furthermore, the Chinese cultural construct, group conformity, can be successfully applied in predicting knowledge purchase intention of China's younger generation. The results show that group conformity has a significant indirect influence on purchase intention in Chinese cultural context while it can directly influence perceived ease of use and perceived usefulness (H6 and H7). Therefore, analyzing cultural influence in this study is not only reasonable but necessary to understand knowledge purchase intention of Chinese younger generation (Zhou et al., 2008; Jiacheng et al., 2010). Our analysis finally reveals that China's younger generation's purchase intention of OPK is influenced by Group Conformity. Thus, they appear to have more tendency to conform to group's choices during the process of using OPK.

## Conclusion and Implications

The primary purpose of the present research was to provide a better understanding of the main factors that influence OPK purchase intention of young Chinese customers. Based on the extended TAM, we proposed a new conceptual framework to fit with the Chinese context. We also explored the interrelationships between perceived usefulness, perceived ease of use, attitude, perceived risk, group conformity, and OPK purchase intention.

### Implications for Research

Theoretically, this study confirms the feasibility of the TAM model to explain certain aspects OPK purchases. Perceived ease of use is positively associated with perceived usefulness and attitude, and the same is true for perceived usefulness and attitude. The results also show that perceived usefulness and attitude are positively correlated with purchase intention. Thus, the results in our study are consistent with the conceptual model proposed in the existing literature (Davis, 1986). Importantly, perceived ease of use is found to be the most significant factor predicting purchase intention while this mainly comes from its indirect influence, which is only sparsely mentioned in the existing literature. These results can be attributed to the significant effects of perceived ease of use on perceived usefulness and attitude (Abdullah et al., 2016). Therefore, the insights offered by this research provide an evidence base for extending the TAM when applied to OPK.

This study also contributes to a better understanding of the factors that influence purchase intention of knowledge by adding and empirically testing two new external constructs—perceived risk and group conformity. Our results reveal that perceived risk has a significant, although indirect effect on customer intention. The risks consumers perceive is an important variable to measure because it can inhibit their evaluation and adoption of OPK (Featherman and Pavlou, 2003). When the level of perceived risk increases, the purchase intention of consumers decreases (Chiu et al., 2014). These are aligned with the findings in our study. Thus, the conditional relationship between perceived risk and the traditional TAM in this study helps to better understand consumer behavior in the field of the sharing economy.

Most importantly, this study examines the influence of a Chinese cultural construct in the form of group conformity and its influence on purchase intention in the Chinese cultural context. The results indicate that consumers in China appear to have a tendency to conform to group perceptions and choices in the use OPK. Such results could be attributed to traditional Chinese culture, where the group plays an important role in dealing with social economic relations (Li and Su, 2007). Therefore, analyzing the influence of culture in this study is necessary to understand the current young generation of Chinese people (Zhou et al., 2008). It extends on the existing research on OPK that has yet to take cultural influence into consideration.

### Implications for Practice

Practically, the research findings have implications for how knowledge platforms are configured and managed by pointing to the allocation of resources to retain and expand their current customer base. Like unpaid knowledge providers, knowledge payment platforms need to strengthen the perception of content usefulness by providing users with a better experience, and creating high-quality knowledge payment products. Platforms need to strengthen their talent support by drawing on the expertise of high-level knowledge contributors ensuring stricter controls over the quality of their content. Second, since perceived ease of use is still considered a significant factor when it comes to the influence over the purchase intention of young Chinese customers, to a certain extent, it could be useful for platforms to enhance the willingness to pay for knowledge by improving the perception of ease of use with more concise page and basic operation logic. It is also vital to provide readily available assistance for consumers when they encounter problems. Third, due to the importance of effective control of perceived risks, managers need to pay sufficient attention to the building of secure firewalls to avoid intrusion, develop methods to enhance private encryption, avoid consumers obtaining useless, redundant or inappropriate information and knowledge, and ensure the safety of the transaction processing servers on their platforms. Fourth, in the Chinese context, the cultural construct, group conformity, is a crucial factor in understanding the willingness of China's young generation to pay for knowledge. Therefore, platforms can emphasize the significant impact of interpersonal interactions, brand awareness, and public praise effects in designing marketing strategies. For example, managers could invite and retain good or well-known knowledge contributors from different fields, like society celebrities and experts, to attract consumers.

### Limitations and Further Research Directions

Despite the contributions this makes, it is not without limitations. First, the survey samples were limited to college students in China. Considering the cognitive differences across cultures, future research should look to continue conducting cross-cultural comparisons. Second, college students' purchase intention of OPK reflects only one segment of China's young generation. Future research could look to analyse older Chinese generations to understand the nature of this market. Future research may analyze other groups in China to understand the nature of this market, not just the younger generation.

In addition, some attribute information may be incomplete or inaccurate when dealing with complex decision-making problems. For different decision-making problems, decision-makers may choose different fuzzy sets to portray uncertain information. Pythagorean fuzzy sets can effectively describe the uncertainty of things (Wang et al., 2021). On this basis, we believe that Pythagorean fuzzy interactive Hamacher power aggregation operators for assessment of consumers' purchase intention with entropy weight have certain feasibility and a higher effectiveness, which is worthy of further digging.

## Data Availability Statement

The raw data supporting the conclusions of this article will be made available by the authors, without undue reservation.

## Author Contributions

WL and ZC conducted the modeling and data analysis and drafted of the manuscript. AX and YZ conceptualized the study, designed the experiment, and contributed to the manuscript. SZ and L-AC contributed to the acquisition and computation of data. All authors critically revised the manuscript for important intellectual content and approved the final version.

## Conflict of Interest

The authors declare that the research was conducted in the absence of any commercial or financial relationships that could be construed as a potential conflict of interest.
